# The effectiveness of an attention‐based intervention for school‐aged autistic children with anger regulating problems: A randomized controlled trial

**DOI:** 10.1002/aur.2800

**Published:** 2022-08-31

**Authors:** Pamela Clifford, Carolien Gevers, Kim M. Jonkman, Frits Boer, Sander Begeer

**Affiliations:** ^1^ Wei43 Amsterdam KN The Netherlands; ^2^ Department of Clinical‐Neuro and Developmental Psychology Vrije Universiteit Amsterdam Amsterdam BT The Netherlands; ^3^ Amsterdam Public Health Vrije Universiteit Amsterdam Amsterdam BT The Netherlands; ^4^ Child and Adolescent Psychiatry Amsterdam Medical Center Amsterdam AZ The Netherlands

**Keywords:** Affect/emotion, Behavioral intervention, Children, Clinical Trials, Intervention, Treatment research

## Abstract

Anger regulation is a challenge for children with autism spectrum disorders (ASD). We investigated if attention‐based cognitive behavioral treatment, based on mindfulness cognitive therapy (MBCT) and dialectical behavior therapy (DBT), reduces aggressive behavior and improves anger coping in school‐aged autistic children (*n* = 51). Children were randomized to an active‐control or a treatment condition. The treatment included nine weekly sessions attention‐based individual therapy. Parents in both conditions received three weekly psychoeducation group sessions to heighten awareness of expressed emotion (EE). For aggressive behavior, treatment reduced temper tantrums and arguing. No effect was found on destroying things and physical violence. For anger coping, treatment increased adaptive coping strategies of diffusion and social support seeking, but had no effect on assertion, rumination, and maladaptive coping direct anger out and avoidance. Treatment did not impact secondary outcome measures concerning children's quality of life (QoL) and parental stress‐levels and psychological well‐being. In conclusion, school‐aged autistic children are able to acquire self‐regulation skills reducing temper tantrums and arguing and increasing the use of adaptive anger coping strategies. The intervention shows potential to improve behavior and regulation, but little transfer to other domains. Limitations and future directions involving the child's social environment, including parents, siblings, and teachers are discussed.

**Lay Summary:**

Children on the autism spectrum often show aggressive behavior. Treatment can train children to be more aware of their emotions. This study found that this can help reducing temper tantrums and arguing and increasing some coping skills, though no impact was found on several other domains of aggression and coping.

## INTRODUCTION

Aggressive behavior problems are estimated to occur in 25% to 68% of children and adolescents with autism spectrum disorders (ASD) (Hill et al., 2014; Kanne & Mazurek, 2011). Treatments for aggression in ASD commonly include behavioral interventions. However, recent evidence suggests that a focus on the awareness of anger may also be effective. In this study we describe the effects of treatment for aggression in children with ASD, based on a combination of dialectical behavior therapy (DBT); focused on shifting and controlling attention (Linehan, 2014) and mindfulness based cognitive therapy (MBCT); focused on skills that increase awareness (Baer, 2014; Segal et al., 2013) on children's aggression and use of anger coping strategies.

Aggression in autistic children is typically triggered by withholding preferred behaviors or activities (Kanne & Mazurek, 2011; Reese et al., 2005; Samson et al., 2014; Sofronoff et al., 2007), and amplified by aggressive rumination (Patel et al., 2017), which can co‐occur with hostility, verbal‐ and physical aggression (Ibrahim et al., 2019). Tension within the family system adds to these typical autistic anger‐eliciting factors (Bader & Barry, 2014). The attitude of family members toward an individual, referred to as Expressed Emotion, EE, can be critical, hostile, or overprotective in nature. The critical and hostile variants of EE, when, for example, parents keep telling the child “You never listen!” or “You are selfish!,” relate to increasingly severe externalizing problems over time in autistic children (Bader & Barry, 2014). This can be a heavy burden for family members: behavior problems and temper tantrums are linked to high levels of stress in mothers of autistic children (McStay et al., 2014).

Historically, anger and aggression treatments in autistic children did not directly target self‐regulation, but relied on reinforcement techniques, and pharmacological interventions. This was found to improve behavior, but only in specific contexts. Moreover, medication such as risperidone and aripiprazole can be effective, but comes with significant adverse side effects (Carr & Horner, 2007; Fung et al., 2016; Goel et al., 2018; Wong et al., 2015). Targeting self‐regulation may be an efficient and less intrusive strategy to reduce aggressive behavior. Self‐regulation is an important skill since it helps autistic children to attain their goals independently from external reinforces and across different situations (Singh et al., 2011). Regulating an emotional state like anger, compromises a situation (e.g., “My brother sits in my favorite chair”),*—*attention (e.g., “I keep watching him and keep telling him he has to leave”)—appraisal (e.g., “Him not leaving proofs he is my arch enemy”)—response (e.g., “I am gonna push him away, cause it's my chair”)—sequence. A person can influence his or her emotions through any component of this sequence (Gross, 2015). Where classic cognitive‐behavioral programs for autistic children focus strongly on *appraisal*, aiming to alter dysfunctional thought patterns (Chalfant et al., 2007; Roeyers et al., 2011; Sofronoff et al., 2007; White et al., 2009; White et al., 2010; Wood et al., 2009), CBT approaches such as MBCT and DBT, focus mainly on *attention*, by choosing a new attentional focus within a difficult situation.

Autism is linked to rigidity and reduced perspective‐taking skills. Children with ASD often show difficulty in the spontaneous and independent generation of cognitive reappraisal strategies (Conner et al., 2019; Luiselli, 2014; Samson et al., 2015). Moreover, when an autistic child is on the verge of a temper tantrum this calls for simple tools. These tools might include some of the essential components of both DBT and MBCT: awareness of one's emotional state, moving away from the anger‐provoking situation, shift attention to less aversive stimuli and modulate the response by applying self‐soothing methods (Baer, 2014; Linehan, 2014; Segal et al., 2013). Interventions based on DBT and MBCT seem promising in reducing emotion dysregulation problems and symptoms of depression and anxiety in the adult ASD population (Hartmann et al., 2019; Spek et al., 2013) and improve externalizing problems, attention problems, emotional well‐being, emotion regulation strategies, depression, and anxiety in children (Conner et al., 2019; Ridderinkhof et al., 2018; Tanksale et al., 2020). To date, however, only one small‐scaled study (*n* = 3), using foot sole‐meditation effectively as a way of shifting attention, was targeted directly at aggression (Singh et al., 2011).

In the current study, we investigated the effectiveness of an attention‐based intervention tailored to aggressive behavior problems and the use of anger coping strategies of school‐aged autistic children with anger regulation problems. Using a randomized controlled trial (RCT), children were allocated to the attention‐based treatment in combination with parental psychoeducation group sessions to heighten awareness of EE (treatment group) or to the same parental psychoeducation only (active control group). Expected primary treatment effects were a reduction of aggressive behavior problems and an increase in the use of adaptive anger coping strategies as well as a decreased use of maladaptive anger coping strategies. Expected secondary effects were an improvement of the child's emotional, behavioral functioning and quality of life (QoL), a reduction of parental stress, and an increase in their psychological well‐being. Informants were children, parents, and teachers.

## METHOD

### Participants

Fifty‐one children aged 8–13 years, were included in this study during the time of treatment at two centers for child mental health: *De Bascule* and *Praktijk Wei43*, both based in Amsterdam, The Netherlands. The majority of them were Dutch and came from middle to high‐SES households (Table 1). All children attended schools that require average IQ levels. Although the sample showed some ethnic diversity, children from immigrant families were underrepresented. This is typical for children receiving mental health services in the Netherlands (Verhulp et al., 2013).

**TABLE 1:** Highest educational level parents, ethnicity, and nationality

Country of birth mother (*n*, %)	
The Netherlands	38 (74.5)
Other	4 (7.8)
Unknown	9 (17.6)
Country of birth father (*n*, %)	
The Netherlands	36 (70.6)
Other	6 (11.8)
Unknown	9 (17.6)
Education mother (*n*, %)	
Low	1 (2.0)
Middle	12 (23.5)
High	27 (52.9)
Unknown	11 (21.6)
Education father (*n*, %)	
Low	3 (5.9)
Middle	14 (27.5)
High	21 (41.2)
Unknown	13 (25.5)
Nationality child (*n*, %)	
Dutch	41 (80.4)
Other	1 (2.0)
Unknown	9 (17.6)

All children had a primary diagnosis of ASD and were not excluded if comorbid attention deficits such as ADHD were present, unless psychotropic medication was still being set. ASD was diagnosed by experienced clinicians, using a developmental history, a child psychiatric examination, an interview with the parents, and a school observation. In case of doubt, the child was assessed with the Autism Diagnostic Observation Schedule (ADOS; Lord et al., 2001). The aggressive behavior problems were (one of) the reason(s) the child was referred to the treatment center.

### Procedure

**FIGURE 1:**
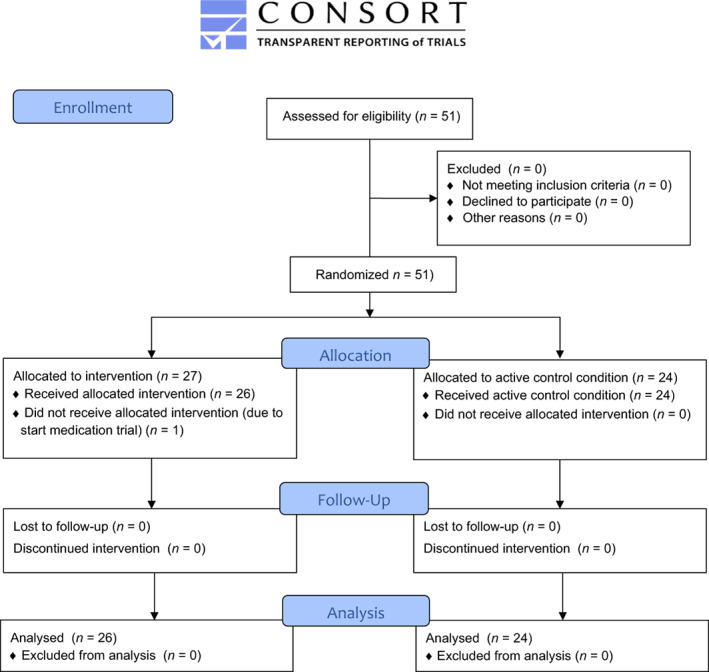
CONSORT 2010 flow diagram of participant flow through the study

Approximately 270 children are annually referred to the Bascule's outpatient clinic for ASD and ~10 children with ASD to *Praktijk Wei43*. All children included were recruited between January 2011 and October 2018. Children were randomly assigned to the intervention or control condition as consent forms were returned. An independent researcher randomized children using a digital random number generator. The randomization outcome was shared with the study coordinator, who informed patients about allocation outcome, see flowchart Figure 1.

The intervention group received the individual intervention, “Anger Can Go!” (Gevers & Clifford, 2009), immediately after the parents finished their three parental psychoeducation group sessions. The control group received the intervention after 9 weeks. Their parents, however, followed the same parental sessions. Therefore, the children in the control group were measured nine weeks after the parental sessions were over, as were the children in the treatment group. The therapists who treated the children and their parents, were all psychologists trained to apply the “Anger Can Go!” program by senior clinical psychologists of the two treatment centers. Supervision‐sessions were included to ensure treatment fidelity.

### Treatment procedure

The intervention “Anger Can Go!” (Gevers & Clifford, 2009) was designed to treat anger regulation problems in autistic children aged 8 to 13 years old. The intervention consists of nine sessions of 60 min and is divided in four phases. Phase 1 (Sessions 1, 2): psychoeducation, affect‐education, and measuring anger with an anger‐thermometer. This is a self‐report scale presented as the drawing of a thermometer, that allows the child to indicate his level of anger, as linked to specific bodily and behavioral representations on a scale from 0 to 3. Phase 2 (Sessions 3, 4): making a functional behavior assessment (FBA) and taking a time‐out at a low anger level (between 1 and 2 on the scale 0 to 3) to prevent aggressive outbursts. Phase 3 (Sessions 4, 5, 6, 7, 8, 9): taking a time‐out at a low anger level (between 1 and 2 on the scale 0 to 3) to prevent aggressive outbursts, shifting attention away from aversive stimuli, to cope with the stress of the anger‐provoking situation. Phase 4 (Sessions 8, 9): creating solutions to cope with an anger‐provoking situation.

Elements from DBT (attention control, distraction, and shifting attention away from aversive stimuli) and MBCT (foot sole/walking meditation, drawing meditation, watching‐meditation, listening meditation, and separating facts from judgements) and shifting attention to trivializing self‐talk (e.g., “It's no big deal, next time I could win,” “My brother means no harm, he's just a toddler”) are trained during Phase 3 and 4 to make shifting attention and hence diffusion easier (Baer, 2014; Dodge & Petit, 2003; Linehan, 2014; Segal et al., 2013). Anger‐thermometer registrations are made on a daily basis. Importantly, the time‐out is not used as a punishment procedure forced upon the child by the parents or teachers, but is a self‐regulation tool. During time‐out the child engages in mindful activities also called concentration exercises, that elicit positive feelings that calm him down and may coincide with his preoccupations.

In the intervention, a boy named Bob acts as a protagonist and role model, he is a child that quickly becomes angry. The sessions are centered around stories and pictures about Bobs aggressive behavior. Bob is not always having anger management problems, but has “ordinary moments,” when he is happily engaging in his hobbies. By having the child register its own “ordinary Bob‐moments” throughout the program, it becomes aware of its competence and its relaxed moments.

Sessions are highly structured using a workbook that contains detailed, literary descriptions of all the exercises and session‐themes and (one of) the parents are present during the last 10 min of the session. Parents are given an outline of the session told by the child and are informed about the homework assignments. Therapists visit school to introduce and practice the time‐out procedure with teachers.

Three parental psychoeducation group sessions to heighten awareness of EE take place before the children's sessions. Parents meet with other parents and a therapist to learn about the nature of their EE and how it relates to the child's aggressive behavior. A detailed description of session outlines is given in Appendix B.

### Design

The study was a randomized controlled trial where the intervention group received both the parental psychoeducation sessions and the anger control treatment, while the control group received only the parental sessions. This allowed for an active control group design, as the parents had information to work with while they waited for the treatment to start. The pre‐treatment measures were collected before the parental sessions started. The Project was approved by the Medical Ethics Committee of the VU University Medical Center (Project No. 2010/241). The study was preregistered at the OSF/Center for Open science (https://osf.io/t2v8z/ ), and the trial was registered at the Dutch Trial Register prior to the recruitment start (www.trialregister.nl), Trial NL2583 (NTR2708), ClinicalTrials.gov Identifier: NCT05221515, also see the Consort Checklist in the supplementary materials section.

### Measures

Measured variables (all pre‐ and posttest) consisted of child‐, parent‐ and teacher‐rated outcome measures.

#### Primary outcome measures

##### Aggressive behavior problems

**QSB**: The Questionnaire Social Behavior (QSB), is a parent or teacher questionnaire especially developed for this study with items focused on behavioral and emotion regulation problems typical for autistic children. The scale “Aggressive Behavior Problems” comprises the subscales Arguing, Temper tantrums, Destroying things, and Physical violence and was used to identify the frequency, intensity, and duration of the various aggressive behavior problems. The internal consistency of the QSB was calculated for the mother, father, and teacher. For both parents the QSB had excellent reliability (Cronbach's alpha parents = 0.91), for the teacher the reliability was rather questionable (Cronbach's alpha teacher = 0.68).

**CBCL**: The Child Behavior Checklist (CBCL; Verhulst et al., 1996). The CBCL is a checklist filled out by parents, to identify emotional and behavioral problems in children and adolescents. The 2001 revision of the CBCL/6–18 (original version in English) has good test–retest reliability and internal consistency and strong criterion‐related validity (Achenbach & Rescorla, 2001). The CBCL contains eight syndrome scales: anxious/depressed, depressed, somatic complaints, social problems, thought problems, attention problems, rule‐breaking behavior, and aggression. In this study, the scale aggression was used as a primary outcome measure (Achenbach & Rescorla, 2001).

**TRF**: Teacher rated outcome measures were obtained by the Teacher Rating Form, a teacher version of the CBCL (TRF; Achenbach & Rescorla, 2001). The 2001 revision of the TRF (original version in English) has good test–retest reliability and internal consistency and strong criterion‐related validity. In this study, the scale of aggression was used as a primary outcome measure (Achenbach & Rescorla, 2001).

##### Anger coping strategies

**BARQ‐C**: The behavioral anger response questionnaire for children, the BARQ‐C, (Miers et al., 2007) was used to establish anger coping strategies. Both the parent and child version of this measure were used. There are six BARQ coping strategies. Two of them are considered *maladaptive*: Direct anger‐out (expressing anger aggressively), avoidance (react passively and/or suppress anger). Three are *adaptive*: Assertion (constructively express one's anger or create a solution), diffusion (deflecting the anger to another stimulus or activity), and social support‐seeking (finding support from a friend or relative). The sixth BARQ strategy, Rumination, taps the tendency to cope with one's anger by repeatedly deliberating over its cause. The BARQ‐C, demonstrated good internal consistency and acceptable construct validity (Miers et al., 2007).

##### Secondary outcome measures

**PEDS QL**: Quality of life (QoL) was measured by the pediatric quality of life inventory (PEDS QL; Varni et al., 2001). The PEDS QL consists of four scales: physical functioning, emotional functioning, social functioning, school functioning. Internal consistency reliability for the Total Scale Score and Psychosocial Health Summary Score were acceptable for group comparisons (Varni et al., 2001). This questionnaire was filled out by the children.

**SRS**: The Social Responsiveness Scale (SRS; Constantino & Gruber, 2005) was used to identify the presence and extent of social impairment due to autism symptoms in the child (Roeyers et al., 2011). Reliability nor construct validity are acceptable, and criterion validity is excellent (Egberink et al., 2012). This questionnaire was filled out by the parents.

**NOSI‐K**: Parental stress levels related to raising an ASD child with anger regulation problems, were established by The Nijmeegse Ouderlijke Stress Index‐K, the Parent Domain. The NOSI‐K is the short, Dutch version of the PSI, Parental Stress Index (de Brock et al., 1992). The NOSI‐K is designed to identify which potential sources of stress within parenting situations actually are experienced as stressful. For the present study, the internal consistency, as assessed by Cronbach's alpha was 0.93 for the Parent Domain of the NOSI‐K/PSI.

**SCL‐90**: The Symptom Checklist‐90 (SCL‐90; Arrindell & Ettema, 2005), a relatively brief self‐report questionnaire, is designed to evaluate a broad range of psychological problems and symptoms of psychopathology in adults. It was used to look into parental symptoms of psychopathology and hence, well‐being. The reliability is acceptable/sufficiently, the construct validity is good, and the criterion validity is good .

### Statistical analysis

All analyses were done using IBM SPSS Statistics (version 26). Baseline differences in demographic variables were tested using chi‐square tests and analyses of variance. To test the effectiveness of the intervention, a multiple linear regression with posttest scores as the dependent variable was used. First pretest scores were added as an independent variable and then treatment group was added to the model. The change in *R*‐squared after adding the treatment group into the model was used as the effect size. Primary and secondary outcomes were corrected for multiple testing using the Bonferroni‐correction. The new levels of significance were 0.05/10 = 0.005 and 0.05/4 = 0.0125 (shown in the result tables) as 10 and 4 scales were tested.

## RESULTS

### Preliminary analyses

Chi‐square tests and analyses of variance were used to test baseline differences in demographic variables (age, sex, and diagnosis). No significant differences between the intervention and control group on these variables were found, nor were there significant differences between intervention and control group in measures concerning ethnicity and SES of the mothers, nor in SES of the fathers. Between the intervention and control group was a significant difference in country of birth of the fathers, but in both groups the majority of fathers were born in The Netherlands. The descriptive information of these demographics is shown in Table 1 and Table 2. Table 7 shows the average baseline scores and standard deviations for all outcome measures. Table A1 shows the correlations among the predictor variables (Appendix A).

**TABLE 2:** Baseline demographics of the treatment and control group

Variable	Treatment (*N* = 26)	Control (*N* = 24)
Age		
N (Nmiss)	26 (0)	24 (0)
Mean (SD)	10.2 (1.58)	10.2 (1.56)
Min–max	7.5–13.9	7.9–12.9
Sex (%)		
N (Nmiss)	26 (0)	24 (0)
Female	23.1	25.0
Male	76.9	75.0
SRS (%)		
N (Nmiss)	21 (5)	19 (5)
Above cut‐off	90.5	94.7
Diagnosis (%)		
N (Nmiss)	25 (1)	23 (1)
PDDNOS	69.3	62.5
Asperger	26.9	25.0
Autism disorder	0.0	8.3

### Primary outcomes

#### Aggressive behavior problems

Aggressive behavior problems were reported by parents and teachers using the QSB. The results of the linear regression to estimate the effectiveness of the intervention on aggressive behavior problems using pretest scores and treatment group as predictor variables, are shown in Table 3. The intervention did not have a significant effect on the Aggressive Behavior Problems scale as a whole, as reported by the parent (*R* ^*2*^ = 0.11, *p* = 0.036). Further analyses of the Aggressive Behavior Problems subscales showed a significant reduction in Temper tantrums (*B =* −2.84, *p* = 0.004, *R* ^*2*^ = 0.20). The subscale Arguing was significantly reduced as well (*B* = −1.60, *p* = 0.026, *R* ^*2*^ = 0.13). The subscales Destroying things and Physical violence showed no significant improvement (Table 5). Temper tantrums showed a significant reduction in frequency (*B =* −0.939, *p* = 0.015), duration (*B =* −0.857, *p* = 0.024) and intensity (*B =* −0.985, *p* = 0.011). Arguing showed a significant reduction in frequency (*B* = −0.693, *p* = 0.034) and intensity (*B* = −0.612, *p* = 0.029), but not in duration (*B* = −0.309, *p* = 0.213).

**TABLE 3:** Teacher and parent measures on aggressive behavior problems (QSB) and aggression (CBCL and TRF) at pretest and posttest for the treatment and control group

	Means (SD) treatment		Means (SD) control		Treatment effect		
Respondent	Pretest	Posttest	Pretest	Posttest	B	*p*	*R* ^2^
Aggressive behavior problems (QSB)							
Teacher	9.30 (9.18)	7.94 (7.83)	7.57 (9.06)	4.31 (9.33)	1.756	0.364	0.010
Parent	16.23 (7.67)	11.15 (7.38)	16.50 (6.80)	16.31 (7.82)	−5.273	0.036	0.113
Aggression							
Teacher (TRF)	11.88 (10.01)	11.24 (10.00)	7.90 (10.57)	8.60 (12.90)	−1.424	0.392	0.004
Parent (CBCL)	15.81 (6.58)	13.11 (6.21)	14.77 (6.46)	14.96 (6.47)	−2.385	0.210	0.036

There was no treatment effect found in aggression as measured by the TRF and CBCL (Table 3).

#### Anger coping strategies

Anger coping strategies were reported by parents and children using the BARQ‐C (see Table 4). The intervention had a significant effect on the use of adaptive anger coping strategies as reported by the parents (*R* ^*2*^ = 0.17, *p* = 0.001). Children who followed the intervention showed an increase in the use of adaptive anger regulation strategies compared with children who did not follow the intervention. Further analysis on the level of the individual strategies shows that this effect was due to an increase in Diffusion (*B =* 2.834, *p* = 0.002) and Social support‐seeking (*B =* 1.516, *p* = 0.029) (Table 5). Assertion did not improve (*B* = 0.723, *p* = 0.06). The use of maladaptive strategies Direct Anger‐out and Avoidance did not show a reduction, nor did the use of Rumination (Table 5).

**TABLE 4:** Child and parent measures on anger regulation strategies (BARQ) at pretest and posttest for the treatment and control group

	M (SD) treatment		M (SD) control		Treatment effect		
Respondent	Pretest	Posttest	Pretest	Posttest	*B*	*p*	*R* ^*2*^
Adaptive strategies (BARQ)							
Child	31.11 (6.70)	35.57 (8.60)	31.33 (7.65)	32.70 (8.95)	2.816	0.151	0.026
Parent	29.69 (4.96)	33.45 (6.01)	30.87 (5.27)	28.84 (4.13)	4.637	0.001^a^	0.171
Maladaptive strategies (BARQ)							
Child	26.28 (3.61)	26.40 (4.42)	27.66 (3.63)	27.00 (4.52)	0.564	0.573	0.004
Parent	27.00 (3.92)	26.32 (3.54)	26.89 (3.94)	26.31 (4.68)	−0.064	0.953	0.000
Rumination (BARQ)							
Child	9.48 (2.95)	8.79 (3.06)	8.19 (2.98)	7.71 (2.53)	0.216	0.732	0.001
Parent	11.41 (2.40)	10.36 (3.02)	11.05 (3.26)	10.58 (3.40)	−0.519	0.443	0.007

^a^ Significant at *p* < 0.005.

**TABLE 5:** Treatment effect on subscale level of parent measures on aggressive behavior problems (QSB) and anger regulation strategies (BARQ)

	Treatment effect		
Subscale	*B*	*p*	*R* ^*2*^
Aggressive behavior problems (QSB)			
Temper tantrums	−2.84	0.004^a^	0.20
Arguing	−1.60	0.026^a^	0.13
Destroying things	0.53	0.396	0.02
Physical violence	−1.35	0.110	0.07
Adaptive strategies (BARQ)			
Diffusion	2.83	0.002^a^	0.17
Social support‐seeking	1.52	0.029^a^	0.07
Assertion	0.72	0.060	0.07
Maladaptive strategies (BARQ)			
Anger	−1.25	0.197	0.02
Avoidance	0.76	0.242	0.03

^a^ Significant at *p* < 0.05.

No effect was found for adaptive strategies nor the use of maladaptive anger coping strategies Direct Anger‐out and Avoidance or Rumination as reported by the children themselves.

### Secondary outcomes

The predicted increase in child or parent well‐being in the intervention group was not found. Children and parents in the intervention and control group had similar improvements in quality of life, social impairment, and parental stress (Table 6).

**TABLE 6:** Child and parent measures on quality of life (PEDS‐QL), social impairment (SRS), parental stress (NOSI) and well‐being (SCL‐90) at pretest and posttest for the treatment and control group

	M (SD) treatment		M (SD) control		Treatment effect		
Respondent	Pretest	Posttest	Pretest	Posttest	*B*	*p*	*R* ^*2*^
Quality of life (PEDS‐QL)							
Child	27.88 (15.87)	28.50 (12.72)	29.22 (11.21)	30.11 (14.61)	−0.713	0.845	0.001
Social impairment child (SRS)							
Parent	82.10 (21.49)	72.42 (19.69)	91.98 (32.57)	81.00 (23.37)	−4.643	0.494	0.011
Parental stress (NOSI‐K)							
Parent	32.38 (10.80)	34.11 (8.33)	28.95 (7.92)	30.50 (8.93)	2.559	0.328	0.021
Psychological well‐being (SCL‐90)							
Parent	135.61 (36.14)	128.08 (21.55)	135.01 (38.21)	121.79 (16.20)	8.886	0.193	0.055

**TABLE 7:** Means and standard deviations at baseline for all outcome measures

Variable	*M*	*SD*
QSB parent		
Temper tantrums	5.79	2.88
Arguing	5.95	2.45
Physical violence	3.30	2.51
Destroying things	1.12	1.79
QSB teacher		
Temper tantrums	3.09	3.67
Arguing	2.77	2.67
Physical violence	1.56	2.46
Destroying things	1.41	2.41
Aggression		
CBCL	15.02	6.46
TRF	10.52	9.99
BARQ		
Assertion	6.88	1.33
Anger	15.77	3.44
Social support seeking	9.49	2.46
Rumination	11.20	2.81
Avoidance	11.29	2.58
Diffusion	13.94	3.34
PEDS‐QL	28.95	12.94
SRS	86.89	27.08
NOSI		
Mother	30.59	9.69
Father	31.33	13.05
SCL‐90		
Mother	136.76	37.15
Father	116.29	23.24

## DISCUSSION

This study investigated whether an attention‐based intervention was effective in reducing aggressive behavior and increasing adaptive anger coping strategies in autistic children. For aggressive behavior, the treatment showed a reduction in temper tantrums and arguing, but not in destroying things and physical violence. Temper tantrums decreased in frequency, intensity, and duration after treatment. Arguing decreased in frequency and intensity, but not in duration. These effects were observed by parents, but not by the teachers. For coping strategies, the treatment improved the adaptive anger coping strategies diffusion and social support‐seeking observed by the parents. However, assertion did not improve and no effect was found on rumination nor on the maladaptive strategies direct anger‐out and avoidance. Children did not report improvements on the use of anger coping strategies. Secondary outcomes including Quality of Life, parental stress, and subjective well‐being were not affected by the treatment.

The reduction of temper tantrums and arguing could be related to the time‐out procedure, which children initiated themselves, and was generally accepted by the children as a rule at low anger levels. This quickly led to de‐escalations. Physical violence and destruction were not affected, but relatively rare in this outpatient population, so floor effects should be considered as well. Still, if physical violence and destruction were present, this occurred at the highest level of anger when children were disturbed during time‐out by siblings. This is in line with findings that autistic children can show destructive behavior during play and often show bad temper and aggression towards siblings (Dempsey et al., 2012; Mascha & Boucher, 2006; Shivers et al., 2019). Including sessions with siblings about the time‐out procedure, could be an important future implication and might have a positive effect on preventing physical violence, destruction, and the duration of arguing.

No treatment related reductions in aggression were found on the CBCL. An explanation may be that the CBCL aggression scale contains many items referring to active oppositional and intentional aggression like “cruelty, bullying or meanness to others,” “threatens other people,” or “teases a lot,” Reducing this kind of behavior was not trained in the intervention, that mainly targeted reactional aggression. Teachers did not see improvements in aggressive behavior problems. In the classroom, the problem may be that teachers seemed quite reluctant to allow children to practice the time‐out as a self‐management coping strategy, since they were used to apply the time‐out as a punishment procedure. Teachers' commitment could be heightened by offering more sessions at school. These matters were not analysed in the current study.

Treatment effects were found for the adaptive anger coping strategies diffusion and social support‐seeking as observed by the parents. Diffusion refers to the ability to deflect one's anger to another object or situation. This was trained intensively, by taking the self‐chosen time‐out, shifting attention, and applying self‐soothing concentration exercises. The intervention allowed preoccupations, like drawing, reading favorite comic books, building with lego, or using play‐doh to coincide with the self‐soothing concentration exercises. Hence, they may have served as a strong motivation for taking the time‐out and deflecting attention. Social support seeking was practiced during role play and homework assignments during the last phase of the intervention when children had to find solutions for anger eliciting situations. Similar to the time‐out procedure, getting help from one's parents and teacher was trained and accepted as a rule at low anger levels. This way, the intervention apparently used autistic features such as rule‐based learning and indulging in preoccupations in a positive manner. Children seemed to be more aware of their anger, but also of their pleasant emotions and how to attain them.

Anger rumination did not improve. Next to the provocative influence of siblings, this might have contributed to the lack of improvement on assertion and the persistence of the maladaptive coping strategy direct anger out. Assertion means talking to the anger provoking person in a calm manner. This requires social skills and letting go of one's hostility. Persevering in anger rumination, is not helpful in accomplishing this. This seems consistent with the results of a longitudinal study that baseline levels of worry/rumination predicted externalizing problems later in life for boys with ASD (Bos et al., 2018). Maybe the use of some of the maladaptive coping strategies is actually adaptive for children with ASD. Spek et al. (2013) did not find a relation between reduction in rumination and a rise in positive affect in ASD after a mindfulness‐based intervention. The authors suggest that rumination might be an autistic coping strategy to control complex social situations, which could be the case for the children in our study. Seen in this way it is interesting that the use of avoidance did not diminish either. Avoidance may be adaptive for autistic children, reflecting the skill of consciously taking a time‐out to regain calmness that was practiced during the intervention.

Eventually, there were no improvements on the child version of the BARQ‐C, measuring anger coping strategy use. Regarding the difference between children and parents, it was found in general that self‐reports of children and parental reports have a low correlation and that some self‐report measures administered to individuals with ASD provide information about their psychiatric condition only to a certain degree (Mazefsky et al., 2010).

The results of our study are in line with other studies showing mindfulness‐based CBT to be effective in improving emotion regulation in autistic children, due to the increase of bodily, emotional and cognitive awareness (Conner et al., 2019; Ridderinkhof et al., 2018; Singh et al., 2011). Furthermore, the results seem congruent with the improvement of executive functioning in autistic children after a CBT program using mindfulness‐based techniques and yoga, resulting in better inhibition, planning, and organization of behavior (Tanksale et al., 2020). In our study parents saw significant improvements in executing adaptive anger coping strategies that have in common moving away from the anger provoking person or situation.

The treatment did not improve QoL. This could be because our QoL measure, the PEDS‐QL, focuses mainly on concrete behavioral aspects and has no separate subscale measuring *psychological well‐being*. Parental stress‐levels were not reduced, nor did their feelings of psychological well‐being improve. Again, coping with parental stress caused by the child's aggression was not specifically trained, but the absence of parental stress reduction may also reflect stress heightening factors, apart from the child's externalizing behavior. These include feelings of loss and grief after the diagnosis (Fernández‐Alcántara et al., 2016), or maternal fatigue, which is known to influence the use of ineffective coping strategies, such as worrying, and increase stress in mothers of autistic children (Seymour et al., 2013; Zaidman‐Zaid et al., 2017). These factors were unexplored in this study. Offering parents a mindfulness training could be a useful addition to the “Anger Can Go!” intervention, since practicing mindfulness results in less worrying and better coping skills in autistic adults and parents of children with ASD (Ridderinkhof et al., 2018; Spek et al., 2013).

The study had several limitations, aside from the relatively small sample. We found no effect of the parental psychoeducation sessions on our primary outcome measures: the child's aggressive behavior and strategy use. It would be interesting though, to see if there is an effect of psychoeducation on levels of EE. This could be analysed in future studies. Furthermore, a teacher version of the BARQ‐C could have given more information about the use of anger management strategies in school settings. Measures of the teacher's adherence to the intervention could have given information about processes influencing effects of the “Anger Can Go!” program in the classroom. The same goes for measuring therapy adherence of parents and siblings. Finally the fact that we constructed the QSB especially for this study could be considered as a limitation.

A major strength of this study is that an RCT design was used. The fact that the sample size was relatively small and follow‐up measures were not taken is a limitation. Furthermore, the demographic homogeneity of the sample limits the generalization of the findings to ASD children from different backgrounds, such as ethnic minorities or low‐SES households.

In conclusion, this study has shown that school‐aged autistic children are able to acquire self‐regulation skills reducing some aspects of aggressive behavior problems, namely temper tantrums and arguing, and improving the use of adaptive anger coping strategies as diffusion and social support seeking. The simplicity of the tools might make the intervention useful for less verbal ASD populations. Extending the program with more sessions with the autistic child and adding extra sessions with parents, siblings, and teachers could create a greater response and give more insight in the dynamics of physical violence, if it occurs.

## Data Availability

The data that support the findings of this study are available on request from the corresponding author. The data are not publicly available due to privacy or ethical restrictions.

## APPENDIX A

**TABLE A1:** Correlations among the predictor variables

	Aggressive behavior problems (QSB) parent	Aggressive behavior problems (QSB) teacher	Aggression (TRF) teacher	Aggression (CBCL) parent	Adaptive strategies (BARQ) child	Adaptive strategies (BARQ) parent	Maladaptive strategies (BARQ) child	Maladaptive strategies (BARQ) parent	Rumination (BARQ) Child	Rumination (BARQ) Parent	Quality of life (PEDS‐QL) child	Parental stress (NOSI) parent	Psychological well‐being (SCL‐90) parent	Social impairment child (SRS) parent
Aggressive behavior problems (QSB) parent	1													
Aggressive behavior problems (QSB) teacher	−0.079	1												
Aggression (TRF) teacher	−0.318	0.760**	1											
Aggression (CBCL) parent	0.521**	0.156	0.012	1										
Adaptive strategies (BARQ) child	−0.415**	−0.173	−0.086	−0.058	1									
Adaptive strategies (BARQ) parent	0.170	−0.212	−0.309	−0.135	−0.037	1								
Maladaptive strategies (BARQ) child	−0.110	0.152	0.018	0.017	0.397**	−0.081	1							
Maladaptive strategies (BARQ) parent	0.086	0.434*	0.346	0.271	0.008	0.157	0.182	1						
Rumination (BARQ) Child	−0.214	−0.036	−0.084	0.043	0.115	−0.055	0.167	−0.074	1					
Rumination (BARQ) Parent	0.308*	−0.484**	−0.362*	0.102	−0.126	0.152	0.025	−0.308*	0.224	1				
Quality of life (PEDS‐QL) child	0.040	0.010	0.113	0.046	−0.210	0.021	0.183	0.036	0.277	0.238	1			
Parental stress (NOSI‐K) parent	0.411**	−0.033	−0.178	0.106	−0.131	0.077	0.198	0.285	0.087	0.257	0.318	1		
Psychological well‐being (SCL‐90) parent	0.289	0.249	0.138	0.036	−0.257	−0.079	0.101	0.220	0.148	0.153	0.325	0.587**	1	
Social impairment child (SRS) parent	0.187	−0.193	−0.319	0.021	−0.083	−0.147	0.350*	−0.106	0.160	0.429**	0.286	0.390*	0.314	1

*Note*: *Correlation is significant at the 0.05 level (two‐tailed). **Correlation is significant at the 0.01 level (two‐tailed).

## APPENDIX B

Session outline “Anger Can Go” and parental psychoeduction

### B.1. Children's program

The program contains four phases. Phase 1 (Sessions 1, 2), Phase 2 (Sessions 3, 4), Phase 3 (Sessions 4, 5, 6, 7, 8, 9), Phase 4 (Sessions 8, 9). The topics for the nine sessions are:

**Session 1**

First basic session about emotion dysregulation. (In the two basic emotion dysregulation sessions both anger and anxiety are analysed, after that, the choice is made to continue with one of the modules [*Anger Can Go* or *Anxiety Can Go*]).

Introduction Bob and his emotion dysregulation problems, introducing ordinary Bob and affect education using pictures and cartoons. Homework: register ordinary Bob moments on a daily basis, making a collage of pictures representing anger and anxiety.

**Session 2**

Second basic session about emotion dysregulation. Introducing the anger‐thermometer and the concept of building up anger gradually, using pictures in which body language is analyzed corresponding with a scale 0, 1, 2, 3 (from neutral to a bit angry, angry to raged). Then the child is asked to show how his own anger thermometer is represented in his body language. The same is done for anxiety.

Homework: register angry and anxious moments on a daily basis using the thermometer scale, register ordinary Bob moments on a daily basis.

**Session 3**

Teaching how to make an FBA (situation‐behavior‐consequence) and combining this with the gradual rise in anger on the thermometer, creating visible behavioral sequences from 0, to 3. Furthermore: emphasizing how anger feels inside the body (but also describing overt, behavioral signs as shouting, kicking, and hitting). Bodily signs become personalized (what are *your* bodily signs?) by drawing them onto a cartoon of a boy or girl.

Introducing the time‐out, which is to be taken when anger is between 1 and 2. Together with parents a time‐out place is selected at home. This is a nice place where the child can engage in activities he likes. Of course, preoccupations and special interests can serve their positive purpose.

Homework: register angry moments on a daily basis using the thermometer scale, register ordinary Bob moments on a daily basis. Practice taking a time‐out whenever anger is between 1 and 2.

**Session 4**

Making a list of anger triggers based on registrations. Learning how to regain calmness in an anger provoking situation using distraction/shifting attention and concentration techniques such as drawing meditation (or molding modeling clay) as tools. Introducing three ways to cool down: (1) distract yourself/shifting attention away (2) use cool downers (trivializations) (3) concentrate on the activity you were engaged in.

Introducing more concentration techniques to be used to regain calmness and shifting attention: footsole/walking meditation, watching meditation, and watching without judgment.

Homework: register angry moments on a daily basis using the thermometer scale, register ordinary Bob moments on a daily basis. Practice taking a time‐out whenever anger is between 1 and 2. Practice concentration exercises. Therapist visits school, to introduce and practice time‐out with teacher.

**Session 5**

Idem.

**Session 6**

Idem.

**Session 7**

A picture story/fairy tale about Bob is read and analyzed, in which he learns to ignore his anger triggers, using the three ways to cool down. Furthermore, this session repeats Sessions 5 and 6.

Homework: ignoring as an Anger Can Go! technique is added, as is “finding Bob‐solutions” for angry‐moments. Furthermore, homework is the same as after Sessions 4, 5, and 6.

**Session 8**

Role play is added. Solutions for difficult situations, containing the child's anger triggers, have to be made up. Furthermore, this session repeats Sessions 4, 5, 6, and 7.

**Session 9**

Last session: farewell, obtaining one's BOB‐certificate. On the certificate is described what the child's anger triggers are, how he can observe 0,1, 2, 3 in his body (language), when the child when has to take his time out, and what his favorite ordinary Bob moments and his favorite concentration exercises are.

### B.2. Parent group sessions

**Session 1**

Informing parents about parental stress and ASD.

Explaining the concept of EE, Expressed Emotion, and how it is related to internalizing and externalizing problems in children with ASD.

Informing the parents about the child's training program and the principles of self‐awareness and teaching them self‐management skills.

**Session 2**

Parents analyze a vignette of a father and son with ASD in a situation where EE is high.

Parents are taught to use a DBT‐based emotion regulation form to describe the emotions, triggers, physical/bodily equivalents, the judgments that are made by this father, the facts they observe in the vignette, and eventually how the situation could have ended without judgments leading to high EE. Parents observe at home their own difficult situations with high EE, using an emotion regulation form, which is an adaptation of the emotion regulation form used in the DBT skills‐training (Linehan, 2014).

**Session 3**

Situations with high EE are presented by the parents in the group.

Advice is given how EE can be kept at low levels.

Parents are informed about the possibility to take a time‐out when EE is starting to rise to prevent them from showing acting out behavior themselves.
